# Herbivore and pathogen effects on tree growth are additive, but mediated by tree diversity and plant traits

**DOI:** 10.1002/ece3.3292

**Published:** 2017-08-11

**Authors:** Andreas Schuldt, Lydia Hönig, Ying Li, Andreas Fichtner, Werner Härdtle, Goddert von Oheimb, Erik Welk, Helge Bruelheide

**Affiliations:** ^1^ German Centre for Integrative Biodiversity Research (iDiv) Halle‐Jena‐Leipzig Leipzig Germany; ^2^ Institute of Biology/Geobotany and Botanical Garden Martin‐Luther‐University Halle‐Wittenberg Halle Germany; ^3^ Institute of Ecology Leuphana University Lüneburg Lüneburg Germany; ^4^ Institute of General Ecology and Environmental Protection Technische Universität Dresden Tharandt Germany

**Keywords:** BEF‐China, biodiversity and ecosystem functioning, climatic niche, functional traits, fungal pathogens, plant–herbivore interactions

## Abstract

Herbivores and fungal pathogens are key drivers of plant community composition and functioning. The effects of herbivores and pathogens are mediated by the diversity and functional characteristics of their host plants. However, the combined effects of herbivory and pathogen damage, and their consequences for plant performance, have not yet been addressed in the context of biodiversity–ecosystem functioning research. We analyzed the relationships between herbivory, fungal pathogen damage and their effects on tree growth in a large‐scale forest‐biodiversity experiment. Moreover, we tested whether variation in leaf trait and climatic niche characteristics among tree species influenced these relationships. We found significant positive effects of herbivory on pathogen damage, and vice versa. These effects were attenuated by tree species richness—because herbivory increased and pathogen damage decreased with increasing richness—and were most pronounced for species with soft leaves and narrow climatic niches. However, herbivory and pathogens had contrasting, independent effects on tree growth, with pathogens decreasing and herbivory increasing growth. The positive herbivory effects indicate that trees might be able to (over‐)compensate for local damage at the level of the whole tree. Nevertheless, we found a dependence of these effects on richness, leaf traits and climatic niche characteristics of the tree species. This could mean that the ability for compensation is influenced by both biodiversity loss and tree species identity—including effects of larger‐scale climatic adaptations that have been rarely considered in this context. Our results suggest that herbivory and pathogens have additive but contrasting effects on tree growth. Considering effects of both herbivory and pathogens may thus help to better understand the net effects of damage on tree performance in communities differing in diversity. Moreover, our study shows how species richness and species characteristics (leaf traits and climatic niches) can modify tree growth responses to leaf damage under real‐world conditions.

## 1 INTRODUCTION

Biodiversity loss is a key driver of global environmental change (Hooper et al., 2012), with considerable effects on ecosystem functioning and human well‐being (Cardinale et al., 2012; Naeem, Duffy, & Zavaleta, 2012). These effects are substantially modified by biotic interactions across trophic levels (Lefcheck et al., 2015; Soliveres et al., 2016). Understanding the relationships between biodiversity and ecosystem functioning (BEF) requires adequate consideration of the trophic complexity of ecosystems (Hines et al., 2015; Soliveres et al., 2016). For example, the dynamics, structure, and functioning of highly diverse forests are considered to be shaped by interactions between plants and higher trophic level organisms, such as herbivores and plant pathogens (Bagchi et al., 2014; Terborgh, 2012). However, we still lack a clear understanding of how many of these interactions are mediated by biodiversity.

Herbivores and pathogens affect tree growth and performance by damaging plant tissues and influencing resource allocation patterns (Bever, Mangan, & Alexander, 2015; Schowalter, 2012). The degree of damage caused by both herbivores and pathogens has been found to depend on tree diversity and thus to be sensitive to biodiversity loss. For example, increasing tree diversity decreased both herbivore and pathogen damage in many studies (Hantsch et al., 2014; Jactel & Brockerhoff, 2007; Rottstock, Joshi, Kummer, & Fischer, 2014; and references therein). This is commonly explained by a decrease in the availability or apparency of host plants to specialized consumers as the number of nonhost plants in a community increases (Castagneyrol, Giffard, Péré, & Jactel, 2013; Root, 1973). However, opposite patterns of increasing damage with increasing tree diversity have also been reported (Kambach, Kühn, Castagneyrol, & Bruelheide, 2016; Nguyen et al., 2016; Plath, Dorn, Riedel, Barrios, & Mody, 2012; Schuldt et al., 2010). Discrepancies among studies could, for example, depend on the degree of host specialization of dominant consumer species (Castagneyrol, Jactel, Vacher, & Brockerhoff, 2014; Zhang et al., 2017). Most of the studies analyzing herbivore or pathogen damage in a biodiversity context have not, however, explicitly tested whether changes in the relationship between damage and diversity have consequences for tree performance (but see e.g. Schuldt et al., 2015; Dillen, Verheyen, & Smit, 2016).

The relationships between leaf damage, tree diversity, and tree performance might further be influenced by interactions between herbivores and pathogens. There is a wealth of studies on facilitative and antagonistic interactions between herbivore and pathogen species. These interactions are either direct (e.g., herbivores as vectors of plant pathogens, or pathogen toxins that influence herbivore feeding; Hatcher, 1995; Kluth, Kruess, & Tscharntke, 2002; Tack & Dicke, 2013) or indirect, plant‐mediated (e.g., induction or suppression of various plant defenses with beneficial or detrimental effects on other herbivores or pathogens; Stout, Thaler, & Thomma, 2006; Biere & Bennett, 2013). Considering the potential importance of such nonadditive effects for plant performance (which, however, still remain poorly studied; Hauser, Christensen, Heimes, & Kiær, 2013; van Mölken et al., 2014) and for plant‐mediated ecosystem functions, it is surprising that there is very little information available on herbivore–pathogen interactions in a BEF context. The few studies we are aware of that have analyzed both herbivore and pathogen damage in relation to changes in tree diversity have not explicitly taken potential interactions between these different groups of organisms into account (e.g., Folgarait, Marquis, Ingvarsson, Braker, & Arguedas, 1995; Setiawan, Vanhellemont, Baeten, Dillen, & Verheyen, 2014).

Finally, previous studies on herbivore–pathogen interactions often revealed species‐specific or trait‐dependent patterns (see Hauser et al., 2013). This indicates that a general understanding of these interactions in a BEF context requires consideration of plant species traits that influence trophic interaction strength and direction. The effects of morphological (e.g., negative effects of leaf toughness) and chemical traits (e.g., positive effects of nutrient content, and negative effects of secondary defense compounds) of host plants may play a particularly important role in this respect, as they determine palatability and susceptibility to damage (Agrawal & Fishbein, 2006; Coley & Barone, 1996; Schuldt et al., 2012, 2014). In addition, larger‐scale range characteristics of plants might influence local damage levels (Schuldt et al., 2012). For example, plant species with a wide niche breadth provide more opportunities for consumer adaptation and diversification (Lewinsohn, Novotny, & Basset, 2005; Miller, 2012), which in turn might lead to higher herbivore or pathogen pressure also on a local scale (Schuldt et al., 2012). Niche characteristics might also be important if potentially stressful environmental conditions at the plant species' range margins make it more susceptible to damage (Fine, Mesones, & Coley, 2004; Maron & Crone, 2006). While the integration of functional traits has led to a better understanding of BEF relationships in recent years (Cardinale et al., 2012; Fichtner et al., 2017), range characteristics have not been tested explicitly as modifiers of BEF relationships so far but might help to provide us with a better understanding of these relationships under real‐world conditions.

In our study, we analyzed the relationships between leaf herbivore damage, foliar fungal pathogen damage, and their (potentially nonadditive) effects on tree growth in a large‐scale tree diversity experiment. Our aim was to assess how diversity loss influences important interactions at higher trophic levels and their impact on tree growth. Moreover, we tested whether variation in trait and niche characteristics among tree species influences these relationships. Our data are based on leaf damage and tree growth surveys on more than 10,000 individual trees in a subtropical tree diversity experiment (Bruelheide et al., 2014), which covers gradients in tree species richness from monocultures to mixtures containing as many as 24 different species. A previous analysis provided insight into the initial effects of tree species richness on herbivory and tree growth relationships shortly after tree planting (Schuldt et al., 2015) but did not consider pathogen damage and its interaction with herbivory. Our study provides new and comprehensive data from a later developmental stage, where the planted trees have grown much larger and started to interact and form a closed canopy. This can have important consequences for our understanding of trophic interaction effects, because biotic interactions are dependent on ecosystem development and need time to establish (Eisenhauer, Reich, & Scheu, 2012; Reich et al., 2012).

Findings from other forest ecosystems indicate that we can expect negative effects of tree species richness on pathogen damage (Hantsch, Braun, Scherer‐Lorenzen, & Bruelheide, 2013; Rottstock et al., 2014). In contrast, and opposing results from temperate forests (e.g., Guyot, Castagneyrol, Vialatte, Deconchat, & Jactel, 2016; Kambach et al., 2016), findings from natural forests in our study region revealed positive effects of tree species richness on herbivore damage (Schuldt et al., 2010). We, therefore, hypothesized that these patterns (1) lead to a negative relationship between herbivore and pathogen damage across the tree species richness gradient and that (2) this negative relationship results in nonadditive effects of herbivore and pathogen damage on tree growth that are mediated by tree species richness. Moreover, we expected that (3) the strength of tree species richness effects on herbivore–pathogen interactions and their effects on tree growth are mediated by traits and niche characteristics of the tree species that influence their palatability and susceptibility.

## 2 MATERIALS AND METHODS

### 2.1 Study site and experimental design

The “BEF‐China” experiment is located close to Xingangshan (29°08′–29°11′N, 117°90′–117°93′E), Jiangxi province, in the south‐east of China. The climate is subtropical, with a mean annual temperature of 16.7°C and a mean annual precipitation of ca. 1,800 mm (Yang et al., 2013). The experiment consists of two sites of ca. 20 ha each (site A & site B, ca. 4 km apart) at an elevation between 100 and 300 m (Bruelheide et al., 2014). In 2009 (site A), and 2010 (site B) trees were planted on a total of 566 plots, each with a size of 25.8 × 25.8 m^2^. Each plot contains 400 planting positions (1.29 m apart) in a regular grid of 20 rows and 20 columns. Out of a pool of 40 native broadleaved tree species, plots were planted with either monocultures or mixtures of 2, 4, 8, 16, or 24 species. On each site, three replicates (based on different species pools) of a random extinction scenario and three replicates (with different species composition) of two nonrandom (direct or trait‐based) scenarios were used to determine the species composition of the mixtures. In the random scenario, tree species of the less diverse mixtures were selected by randomly partitioning the species composition of the most diverse plots into nonoverlapping fractions by means of a bootstrapping procedure. Species compositions in the two nonrandom scenarios were based on local rarity and the specific leaf area (SLA) of the tree species, respectively, with the rarest species or those with the largest SLA being sequentially eliminated with decreasing diversity of the species mixtures. For further details on the planting design, see Bruelheide et al. (2014).

For our analyses, we focused on 300 study plots (excluding plots with additional manipulation of shrub species richness or of seed family richness; see Bruelheide et al., 2014). The study plots comprised monocultures (80 plots) and mixtures of 2 (64), 4 (32), 8 (16), 16 (8), and 24 (4) species following a random extinction scenario, and mixtures of 2, 4, 8, and 16 species (12 plots for each richness level in each scenario) following the two nonrandom extinction scenarios.

### 2.2 Herbivore and pathogen damage

Standing levels of leaf damage were assessed at the end of the main growing season, five years after planting the trees (site A: September 2014; site B: September 2015). We visually estimated damage levels, a standard approach for large‐scale surveys that can yield very accurate data (Johnson, Bertrand, & Turcotte, 2016). Estimates were conducted separately for leaf herbivore and foliar fungal pathogen damage, based on a well‐tested percentage class system (Schuldt et al., 2010, 2015) with six categories (0%, 1%–5%, 6%–25%, 26%–50%, 51%–75%, >75% overall damage. For the statistical analyses, we followed previous studies and used mean values per damage class (i.e., 0%, 2.5%, 15%, 37.5%, 62.5%, 87.5%). Although this decreases the resolution of the data compared to estimates of exact percentages (Johnson et al., 2016), based on our previous field experience we considered this approach the best trade‐off between estimation accuracy and estimation bias when data are collected by several observers. Our survey design distinguishes more precisely between low levels of leaf damage to take special account of the fact that low damage levels occur more frequently than high percentages of leaf damage. To further ensure consistent data quality, observers were trained for 1 week in the field at the beginning of each survey. This training was designed to enable observers to distinguish between different types of herbivore, pathogen, and mechanical damage and to fine‐tune the technique for damage class estimation. Because we were interested in the overall amount of herbivore and pathogen damage, we did not record damage levels separately for different types of herbivory or pathogen damage. However, we noted the damage type responsible for the main damage on each individual tree (chewing, skeletonizing, mining, sucking, or galling damage for herbivory; mildew fungi, rust fungi, necrotic lesions, spot damage for pathogens).

Damage levels were checked on a total of 21 leaves on three branches per tree (seven leaves per branch produced in the current growing season, to allow for adequate comparison across tree species), with separate estimates for herbivore and pathogen damage on the same leaves. Branches were selected in a stratified way to cover upper and lower canopy regions in larger trees and different sides of the canopy. We considered this design sufficient because trees were still relatively small (mean height over all sampled trees = 3.1 m, *SD* = 1.7 m). To ensure that data of each tree species were replicated with several individuals in all mixtures, the number of sampled trees increased from 36 (the central 6 × 6 tree individuals) in monocultures and 2‐species mixtures to 81 (9 × 9 central individuals) in 4‐species mixtures and 144 (12 × 12) in the 8‐ and more species mixtures.

### 2.3 Tree growth

Dendrometric data were collected in separate campaigns on a yearly basis at the end of the main growing season (September–October of each year). For the tree growth analyses we used the data recorded at the time of the leaf damage assessments (2014 and 2015) and those recorded 1 year earlier (2013 and 2014). For each planting position considered in the leaf damage assessments, we measured tree height (total length [cm] from stem base to apical meristem) and ground diameter (average of stem diameter [mm] measurements in two different directions, 5 cm above the ground; Li et al., 2014). From these values, we calculated basal area (π*ground diameter^2^/4) and wood volume (basal area*tree height*0.7, where 0.7 is the cylindrical form factor, which is the ratio of total tree wood volume to the volume of a cylinder that has the same height and diameter as the tree (Li, Kröber, Bruelheide, Härdtle, & von Oheimb, 2017). The cylindrical form factor varies with stem diameter and age, and was set equal to 0.7, which is an average value for young trees (Hess, Bienert, Härdtle, & von Oheimb, 2015)). We calculated relative growth rates (RGR) as the relative increase in wood volume, tree height, and basal area per year as ln(size in year 2/size in year 1) (see Paine et al., 2012).

### 2.4 Plant traits, niche characteristics, and environmental data

The extent of both herbivore and pathogen damage can depend on morphological and chemical characteristics of the host plants that are related to leaf quality and palatability. We, therefore, included in our analyses important plant traits that have frequently been shown to influence leaf damage (Coley & Barone, 1996; Perez‐Harguindeguy et al., 2003; Poorter, de Plassche, Willems, & Boot, 2004; Schuldt et al., 2012). Morphological leaf traits comprised leaf area, specific leaf area (SLA), leaf dry matter content (LDMC), and leaf toughness. Chemical leaf traits were leaf nitrogen (N) content, leaf carbon (C) content, leaf C:N ratio, leaf phosphorus (P) content, leaf polyphenolics content, and leaf tannin content. Traits were measured on sun‐exposed leaves of a minimum of five individuals per tree species following standardized protocols (Pérez‐Harguindeguy et al., 2013). Details are provided in Kröber, Zhang, Ehmig, and Bruelheide (2014) and Eichenberg, Purschke, Ristok, Wessjohann, and Bruelheide (2015).

Likewise, the degree of leaf damage might be influenced by niche properties of the host plants. This could, for instance, be brought about by differences in the preference of herbivores or pathogens for, and adaptation to, widespread vs. rare plant species (Lewinsohn et al., 2005; Schuldt et al., 2012), or because plants might be more susceptible to damage at their range margins (e.g., Bruelheide & Scheidel, 1999). We, therefore, included climatic niche breadth of all tree species and niche marginality at the study site in our analyses. We defined climatic niche breadth as number of occupied climate classes, obtained from a global climate classification based on the 19 “bioclim” variables of the “Worldclim” dataset (http://www.worldclim.org). The global “Worldclim” bioclimatic variables were separately standardized and subsequently divided into equally sized classes. To take into account the increasing ecological impact of differences with decreasing amount of precipitation, all precipitation values were log‐transformed prior to classification. After range standardization (0–1), 20 classes (class width 0.05) per variable were derived. A homogeneous climate class is then defined by an identical combination of class values (nos. 1–20) over all included layer variables. This approach results in a large number of unique climate classes occupied per distribution range but is suitable for deriving unbiased values of climatic niche breadth. Niche marginality was quantified as the distance between the overall mean of the range‐standardized bioclim values of a species' niche (niche centroid) and the mean value of these 19 variables at the experimental site. Subsequently, this distance was weighted by the proportion of climate niche space occupied by a species in the total climate space of all 40 study species (see Schuldt et al., 2012 for details).

Finally, because of the heterogeneous topography of our study site, we used plot‐level data on mean elevation (m), slope (°), and “northness” (cosine‐transformed radian values of aspect) as covariables. Data were obtained from a 5 m digital elevation model (DEM) that was established based on differential GPS measurements when the experiment was started.

### 2.5 Statistical analysis

Data on leaf damage were averaged across the 21 leaves surveyed per tree to obtain mean levels of both herbivore and fungal pathogen damage per tree individual. Data on trees with negative RGR values were removed because they are likely to indicate measurement errors or mechanical tree damage. Likewise, planting positions with dead or missing trees, and positions for which leaf damage and RGR data could not be matched (owing to inconsistencies in the actual tree positions sampled in the two separate measurement campaigns) were excluded. The total number of trees for the analysis amounted to 10,310 individuals.

We further reduced the dimensionality of the leaf traits data by principal components analysis (PCA) to extract the main components of trait variation across tree species. PCAs were run separately for the four morphological and six chemical leaf traits, because we were interested in the relative contribution of morphological and chemical traits to explaining leaf damage and its effect on tree growth. Morphological and chemical PCs were not strongly correlated (Pearson's *r* ≤ .44 in all cases) and therefore provide independent information. The species scores of the first two principal components (PCs) of each analysis were taken to represent interspecific differences in morphological (PC1 morph, PC2 morph) and chemical (PC1 chem, PC2 chem) traits. These axes explained >70% of the variance in trait data in both analyses (Tables S1, S2). PC1 morph represented a gradient of increasing SLA and decreasing leaf toughness, PC2 morph was negatively related to leaf area. PC1 chem was positively related to leaf nutrient content, PC 2 chem was negatively related to leaf phenolics and tannin content (Tables S1, S2).

We used mixed‐effects models with herbivore damage, fungal pathogen damage and the RGRs of the trees as response variables. First, we tested whether herbivore and pathogen damage influence each other in relation to tree species richness (two models with herbivore and pathogen damage, respectively, as response variables). Second, we tested whether herbivore damage, pathogen damage, and tree species richness interact to influence the RGRs (wood volume, height, and basal area) of the trees. In all models, we also tested whether and how potential leaf damage and richness effects might be mediated by leaf traits and niche characteristics.

To account for the hierarchical data structure, we fit tree species identity and study plot identity, nested in tree species composition, as crossed random effects. Fixed effects were pathogen damage (in the herbivore damage and RGR models), herbivore damage (in the pathogen damage and RGR models), tree species richness, the selected PCs of morphological and chemical leaf traits, niche breadth, niche marginality, study site, and the environmental covariables elevation, slope, and northness. Moreover, because RGR values are not independent of initial tree size (Paine et al., 2012), we included the growth data from the year preceding the damage surveys as a measure of initial tree size. We used initial wood volume in the leaf damage models, as it combines height and basal area data, and either initial wood volume, height or basal area in the respective RGR models. We were also interested in potential interaction effects of leaf damage and richness, as well as in how damage and richness effects might be influenced by plant characteristics and initial tree size. We, therefore, included in our models the interactions between tree species richness and herbivore or pathogen damage, as well as the two‐way interactions between these variables and plant characteristics or initial tree size. We also checked whether the inclusion of the extinction scenarios improved model fit by comparing the initial models with and without a factor (and its interactions with other predictors) that distinguished between plots with random and nonrandom extinction. Because this was not the case in any of the models (based on the model AIC values), indicating negligible differences between random and nonrandom scenarios, we ran the models without distinguishing between extinction scenarios.

The initial models with the full set of predictors and their interactions were simplified by sequentially deleting uninformative terms based on the resulting reduction in the AIC value of the model (Burnham & Anderson, 2004). The model with the smallest number of predictors and the lowest global AIC was chosen as the most parsimonious, best‐fit model and then rerun with restricted maximum likelihood (REML) estimation. All response and predictor variables (except the leaf trait PCs) were log‐transformed to optimize normality and homoscedasticity of the model residuals. Subsequently, all continuous predictors were standardized (mean = 0; *SD* = 1) before analysis. All analyses were conducted in R 3.3.1 (http://www.R-project.org) with the package *lmerTest* (Kuznetsova, Brockhoff, & Christensen, 2016). Model R^2^ values were calculated with the package *MuMIn* (Barton, 2016), following the procedure suggested by Nakagawa and Schielzeth (2013).

## 3 RESULTS

### 3.1 Mean damage levels and growth rates

Leaf damage by herbivores was primarily caused by leaf chewers and skeletonizers, which were responsible for the main damage on 79% of all trees. Mean herbivore damage across all trees was 12.0% (±0.1 *SE*), mean fungal pathogen damage was 5.4% (±0.1 *SE*). However, there was pronounced variation among individuals (*SD* herbivore damage 7.9%, *SD* pathogen damage 6.0%), with maximum damage levels of 66.4% for herbivore damage and 75.0% for pathogen damage on individual trees. Tree species with highest mean leaf damage were the deciduous *Ailanthus altissima* (miller) swingle (21.0% ±1.4 *SE* herbivore damage) and *Diospyros japonica* siebold & zuccarini (17.4% ±0.7 *SE* pathogen damage). Species with lowest mean damage were the deciduous *Triadica sebifera* (L.) small (5.6% ±0.2 *SE* herbivore damage) and the evergreen *Schima superba* gardn. et champ. (2.8% ±0.1 *SE* pathogen damage).

Mean wood volume across all species and tree individuals was 4682.1 (±77.8 *SE*) cm^3^, mean height 250.3 (±1.4 *SE*) cm and mean basal area 17.5 (±0.2 *SE*) cm^2^. RGRs were on average 0.68 (±0.005 *SE*) for wood volume, 0.24 (±0.002 *SE*) for tree height and 0.46 (±0.003 *SE*) for basal area. The deciduous species *Acer davidii* franch. had the highest RGR for wood volume (0.99 ± 0.21 *SE*) and basal area (0.72 ± 0.15 *SE*) (mean initial wood volume and basal area were 547.8 cm^3^ ± 132.6 *SE* and 3.42 cm^2^ ± 0.67 *SE*, respectively). The evergreen *Machilus grijsii* hance showed the highest RGR for tree height (0.32 ± 0.09 *SE*; mean initial height was 168.2 cm ± 10.4 *SE*). Lowest RGRs were observed for two deciduous species: 0.26 (±0.02 *SE*) for wood volume and 0.12 (±0. 01 *SE*) for height in *Rhus chinensis* mill. (mean initial wood volume and height were 2126.9 cm^3^ ± 211.4 *SE* and 203.9 cm ± 6.6 *SE*, respectively) and 0.22 (±0.02 *SE*) for basal area in *Melia azedarach* L. (mean initial basal area was 20.34 cm^2^ ±1.95 *SE*).

### 3.2 Drivers of herbivore and fungal pathogen damage

**Figure 1:**
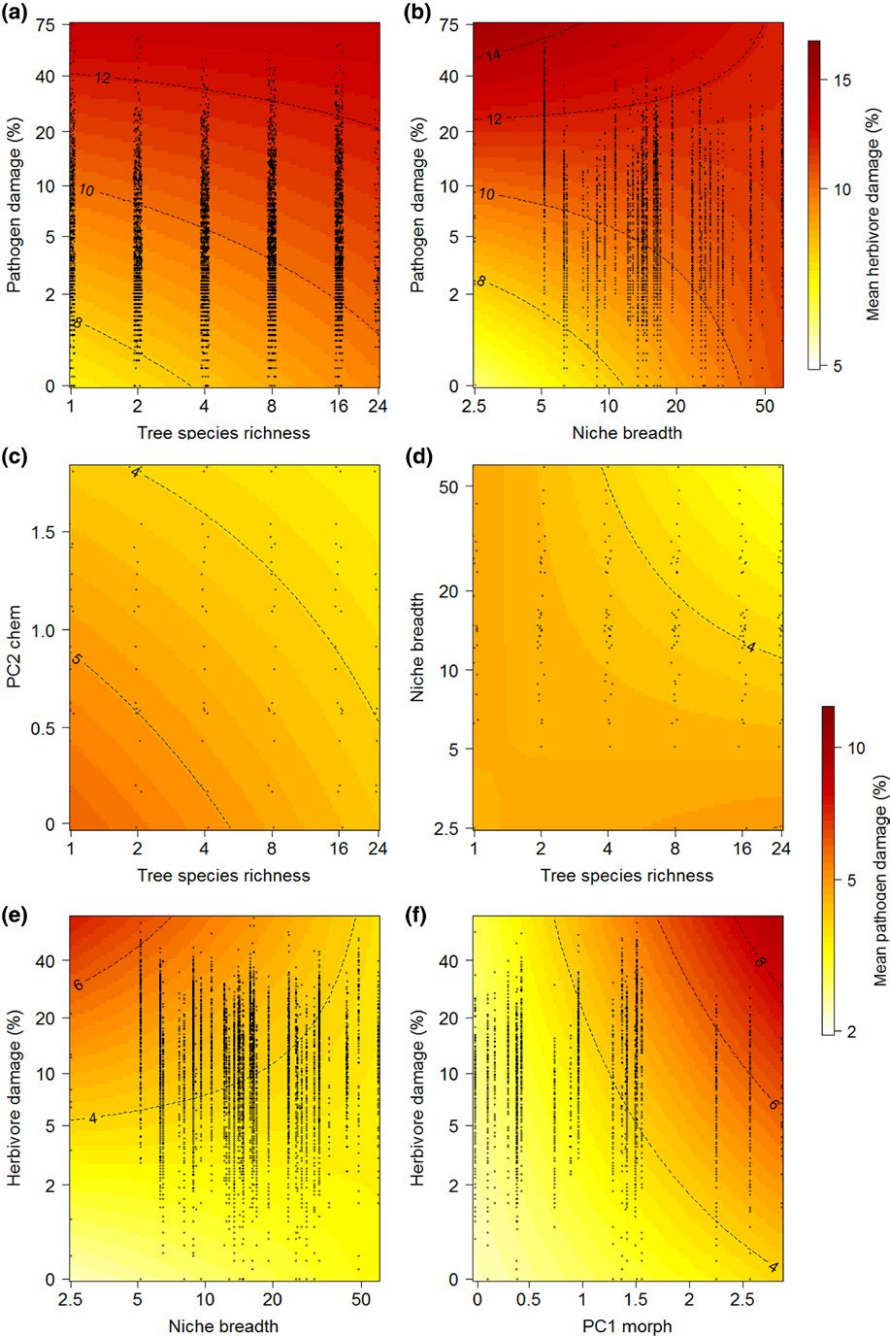
Interactive effects of tree species richness, plant traits, and insect herbivores or fungal pathogens on leaf damage in the “BEF‐China” tree diversity experiment. Note that scales differ for herbivore (a, b) and pathogen (c–f) damage because of differences in overall damage levels between the two groups. Colors and isolines show predicted values of the mixed‐effects models of herbivore and pathogen damage. All interactions were significant at *p* ≤ .05 (see Tables 1 and 2). Black circles show the distribution of observations (jittered for better visualization)

Herbivore damage was significantly related to pathogen damage, tree species richness, and plant traits. However, the strength and direction of these relationships were strongly influenced by interactions among these predictors (Table 1). Most importantly, while herbivore damage was positively related to pathogen damage and tree species richness, pathogen effects on herbivory were most pronounced at low tree species richness, and richness effects were most pronounced when trees showed low pathogen damage (Figure 1a). Effects of tree species richness on herbivory also became stronger with increasing initial tree size (Table 1). Furthermore, pathogen effects on herbivore damage were mediated by the tree species' leaf chemical trait composition (decreasing in strength with decreasing leaf phenolics concentrations of the tree species, PC2 chem; Table 1) and the climatic niche breadth (decreasing in strength from species with narrow niche breadth to species with wide niche breadth; Figure 1b). Herbivore damage also tended to increase with increasing niche marginality of the tree species (Table 1).

**Table 1:** Minimum‐adequate mixed‐effects model for the effects of pathogen damage, tree species richness, plant traits, and plot characteristics on herbivory (*R* ^2^m = 14.3%; *R* ^2^c = 48.5%)

Predictor	Std. Est.	*SE*	*df*	*t*	*p*
(Intercept)	2.05	0.07	38	30.3	<.001
Site B	0.56	0.04	275	13.6	<.001
Elevation (log)	0.06	0.02	220	2.9	.004
Slope (log)	0.04	0.01	231	2.6	.009
Pathogen damage (log)	0.07	0.01	10,100	10.8	<.001
Tree species richness (log)	0.04	0.01	110	2.8	.006
Initial wood volume (log)	0.05	0.01	9,993	6.9	<.001
*PC1morph*	−*0.11*	*0.06*	*30*	−*1.9*	*.070*
PC2morph	−0.11	0.05	30	−2.1	.044
*PC2chem*	−*0.04*	*0.06*	*29*	−*0.6*	*.550*
*Niche breadth (log)*	*0.05*	*0.07*	*32*	*0.7*	*.472*
*Niche marginality (log)*	*0.10*	*0.05*	*30*	*2.0*	*.053*
Pathogen damage:PC2chem	−0.03	0.01	10,120	−3.8	<.001
Pathogen damage:niche breadth	−0.02	0.01	10,090	−3.5	<.001
Tree richness:wood volume	0.02	0.01	6,311	2.7	.008
Pathogen damage:tree richness	−0.01	0.01	6,725	−2.1	.039

Nonsignificant terms retained in the minimal models are italicized; log‐transformed predictors are indicated by (log); colons indicate interactions between two predictors. PC1 and PC2 = scores of the first and second principal component of a PCA on morphological or chemical leaf traits (see Tables S1, S2).

Pathogen damage levels were positively related to herbivore damage levels and negatively to tree species richness (Table 2). Again, these relationships were mediated by interactions with plant traits. Negative effects of tree species richness on pathogen damage were most pronounced for species with high leaf phenolics concentrations (low values of PC2 chem; Figure 1c) and for species with wide niche breadth (Figure 1d). Negative niche breadth effects on pathogen damage were most pronounced at high tree species richness and absent in monocultures (Figure 1d). Similar to the results of the herbivore damage model, the positive relationship between pathogen and herbivore damage was strongest for tree species with narrow niche breadth (Figure 1e), and it became weaker with decreasing leaf phenolics concentrations of the species (PC2 chem; Table 2). In contrast, high SLA and low leaf toughness (PC1 morph) increased the strength of potential herbivore damage effects on pathogen damage (Figure 1f).

**Table 2:** Minimum‐adequate mixed‐effects model for the effects of herbivore damage, tree species richness, plant traits, and plot characteristics on pathogen damage (*R* ^2^m = 13.0%; *R* ^2^c = 46.7%)

Predictor	Std. Est.	*SE*	*df*	*t*	*p*
(Intercept)	1.53	0.07	39	22.8	<.001
Site B	0.22	0.04	304	5.4	<.001
Elevation (log)	−0.04	0.02	219	−2.0	.042
Herbivore damage (log)	0.07	0.01	10,120	10.9	<.001
Tree species richness (log)	−0.05	0.01	208	−3.7	<.001
Initial wood volume (log)	−0.02	0.01	10,020	−2.6	.011
PC1morph	0.16	0.06	32	2.8	.009
*PC2chem*	−*0.06*	*0.06*	*31*	−*1.0*	*.334*
*Niche breadth* (*log*)	−*0.03*	*0.06*	*34*	−*0.4*	*.658*
*Niche marginality* (*log*)	*0.10*	*0.05*	*32*	*2.0*	*.055*
Herbivory:PC1morph	0.02	0.01	10,110	3.2	.001
Herbivory:PC2chem	−0.02	0.01	10,120	−3.2	.001
Herbivory:niche breadth	−0.03	0.01	10,120	−3.7	<.001
Tree richness:niche breadth	−0.03	0.01	2,286	−3.3	<.001
Tree richness:PC2chem	0.01	0.01	1,459	2.0	.046

Standardized parameter estimates (with standard errors, degrees of freedom, *t* and *p* values) are shown for the variables retained in the minimal model. Nonsignificant terms retained in the minimal models are italicized. Log‐transformed predictors are indicated by (log). Colons indicate interactions between two predictors. PC1 and PC2 = scores of the first and second principal component of a PCA on morphological or chemical leaf traits (see Tables S1, S2).

### 3.3 Drivers of relative tree growth rates

Results for all three variants of RGR (wood volume, height, basal area) were qualitatively similar (see Table 3 and Tables S3, S4). In the following, we focus on wood volume because it is an integrative metric based on both tree height and basal area. Despite the significant positive effects of herbivore and pathogen damage on each other in the leaf damage models, the RGR of wood volume increased with increasing herbivore damage, but decreased with increasing pathogen damage (Table 3). There was no significant interaction effect of herbivore and pathogen damage on RGR.

**Table 3:** Minimum‐adequate mixed‐effects model for the effects of pathogen and herbivore damage, tree species richness, plant traits, and plot characteristics on the relative tree growth rate (based on wood volume) (*R* ^2^m = 20.9%; *R* ^2^c = 41.9%)

*Predictor*	Std. Est.	*SE*	*df*	*t*	*p*
(Intercept)	−0.40	0.04	55	−9.5	<.001
Site B	−0.07	0.04	253	−2.1	.034
Slope (log)	0.05	0.01	238	3.7	<.001
Pathogen damage (log)	−0.04	0.01	9,634	−6.5	<.001
Herbivore damage (log)	0.02	0.01	9,638	3.4	.001
*Tree species richness (log)*	−*0.01*	*0.02*	*107*	−*0.7*	*.482*
Initial wood volume (log)	−0.16	0.01	9,542	−23.6	<.001
PC1morph	−0.08	0.04	32	−2.2	.032
PC2morph	0.11	0.03	32	3.7	.001
PC1chem	−0.10	0.03	31	−2.8	.009
*Niche breadth (log)*	*0.02*	*0.04*	*35*	*0.6*	*.551*
*Niche marginality (log)*	−*0.06*	*0.03*	*32*	−*2.0*	*.054*
Pathogen damage:wood volume	0.02	0.01	9,765	3.5	<.001
Pathogen damage:marginality	−0.02	0.01	9,664	−3.2	.002
Herbivory:wood volume	−0.02	0.01	9,739	−2.7	.007
Herbivory:PC1morph	−0.01	0.01	9,722	−2.0	.045
Herbivory:PC2morph	0.03	0.01	9,701	4.2	<.001
Herbivory:niche breadth	0.02	0.01	9,647	3.4	.001
Tree richness:PC1morph	0.03	0.01	1,302	4.1	<.001
Tree richness:niche breadth	−0.02	0.01	1,956	−3.0	.003
Pathogen damage:tree richness	−0.02	0.01	8,318	−3.4	.001

Standardized parameter estimates (with standard errors, degrees of freedom, *t* and *p* values) are shown for the variables retained in the minimal model. Nonsignificant terms retained in the minimal models are italicized. Log‐transformed predictors are indicated by (log). Colons indicate interactions between two predictors. PC1 and PC2 = scores of the first and second principal component of a PCA on morphological or chemical leaf traits (see Tables S1, S2).

**Figure 2:**
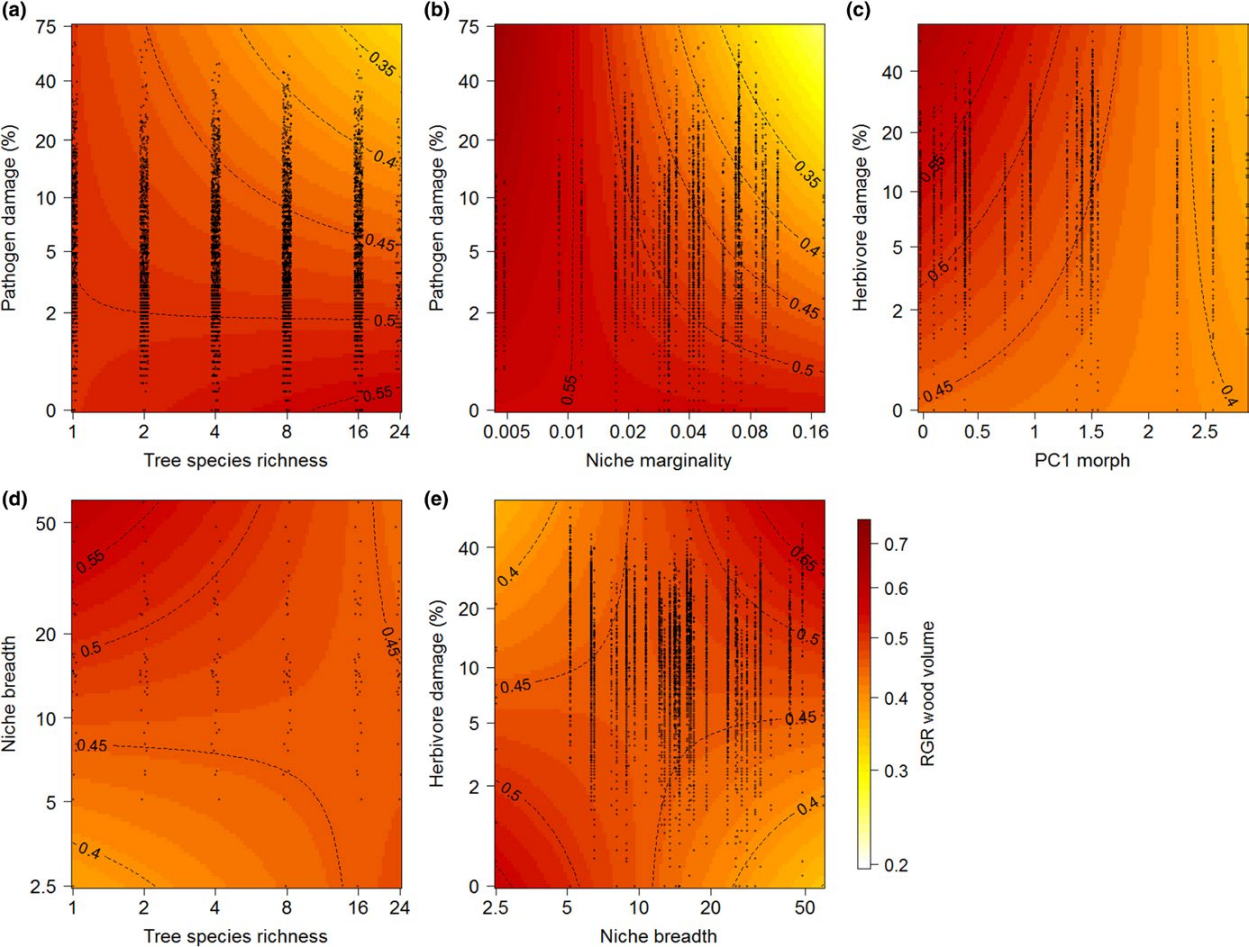
Interactive effects of tree species richness, plant traits, insect herbivores, and fungal pathogens on the relative growth rates (based on wood volume) of the trees planted in the “BEF‐China” tree diversity experiment. Colors and isolines show predicted values of the mixed‐effects model of relative growth rates. All interactions were significant at *p* ≤ .05 (see Table 3). Black circles show the distribution of observations (jittered for better visualization)

However, the negative effects of pathogen damage became stronger with increasing tree species richness (Figure 2a) and increasing niche marginality of the tree species (Figure 2b). Effects of herbivore damage were mediated by tree size (initial wood volume) and morphological leaf traits. The positive relationship between RGR and herbivore damage became weaker with increasing size of the trees, and on trees with high SLA and low leaf toughness (PC1 morph) (Table 3, Figure 2c). RGR decreased with herbivory for species with a narrow climatic niche breadth, but increased with herbivory for species with a wide climatic niche breadth (Figure 2e). The opposite was found for the influence of niche breadth on the effects of tree species richness on RGR: Richness effects were negative for species with wide niche breadth, but tended to be positive for species with narrow niches (Figure 2d). Effects of tree species richness on RGR also depended on leaf toughness and SLA (PC1 morph) and on pathogen damage. RGR increased with tree species richness at low pathogen damage and decreased with richness at high damage (Figure 2a).

## 4 DISCUSSION

Our study shows that despite a positive relationship between herbivore and fungal pathogen damage, the two damage types had contrasting and independent (i.e., additive) effects on tree growth. These damage effects on tree growth were mediated by tree species richness and/or plant characteristics, indicating that biodiversity loss and plant species identity will influence ecosystem responses to herbivore and pathogen damage in distinct ways.

### 4.1 Relationships between herbivore and pathogen damage

The net relationship between herbivores and pathogens may depend on the relative strength of the many possible direct and indirect, facilitative and antagonistic pathways that characterize the interactions between diverse herbivore and pathogen assemblages (Hatcher, 1995; Stout et al., 2006; Tack & Dicke, 2013). Total herbivore and pathogen damages in our study were positively related, which might be indicative of an important role of facilitative direct effects (herbivores as vectors) or indirect effects via plants. Leaf chewers were the main herbivores at our study site (Zhang et al., 2017) and responsible for the main damage on most trees. Leaf chewing herbivores and pathogens such as biotrophic fungi can trigger separate defense pathways that are most effective against only one of the two damage types or that even inhibit alternative defense pathways via signaling cross talk (Thaler, Humphrey, & Whiteman, 2012). However, the positive relationship between herbivore and pathogen damage in our study became weaker with increasing tree species richness (at least the potential effects of pathogens on herbivore damage). This can be explained by the opposite effects of tree richness on herbivory and pathogen damage (see also Hantsch et al., 2014; Schuldt et al., 2010, 2015). Herbivore assemblages in our study system are dominated by generalists (Zhang et al., 2017), which can benefit from the diversity of resources available in more diverse tree communities (Lefcheck, Whalen, Davenport, Stone, & Duffy, 2013; Zhang et al., 2017). In contrast, most foliar fungal pathogens are highly host‐specific and their passive mode of dispersal makes them dependent on the density of suitable hosts in their surroundings (Hantsch et al., 2014), which is highest in monocultures and decreases with increasing tree species richness. This suggests that stand diversification has the potential to disrupt the associations between different types of plant antagonists, at least at the level of whole herbivore and pathogen assemblages. In our case, this led to a weakening of the positive association between herbivore and pathogen damage.

However, this weakening by diversity was only observed for potential effects of pathogens on herbivore damage. Our results indicate that direct or indirect facilitation of herbivory by pathogens could be most effective in monocultures (i.e., in the absence of opportunities for diet mixing), for example because it might help generalist herbivores to overcome tree species‐specific defense mechanisms or alter host traits (e.g., Cardoza, Lait, Schmelz, Huang, & Tumlinson, 2003). The dominant herbivorous arthropods at our study site included many adult leaf chewers, such as weevils (Zhang et al., 2017). These herbivores are highly mobile and can easily move between trees to select suitable host plants. The fact that mobile, generalist herbivores play an important role in determining overall herbivory levels is further supported by the finding that the abundance of adult leaf chewers with a generalized host use increased with increasing tree species richness at our study site, whereas more specialized and less mobile herbivore abundances were unaffected by changes in tree species richness (Zhang et al., 2017). A dependence of facilitative effects on host plant quality would fit the observation that herbivory was positively related to pathogen damage particularly on trees with high tannin contents. At the same time, effects of tree species richness on herbivore damage were strongest when pathogen damage was low. This could mean that richness effects promoting herbivory (e.g., by enabling diet mixing) might be most relevant when there are no additional facilitative effects of pathogens that could help to overcome plant defenses. The important role of leaf traits in determining the interactions between herbivores and pathogens became evident from the fact that herbivory seemed to promote pathogen damage especially on tree species with softer leaves. Previous studies have shown that herbivore damage can facilitate pathogen infection by opening up leaf tissue for pathogen attack (Biere & Bennett, 2013). However, this effect would be expected to be more important for tough leaves, because they might be less easily accessible to pathogens. The fact that we found the opposite patterns could mean that pathogens might be better able to colonize and damage the area surrounding a location predamaged by herbivores on softer leaves.

Besides morphological and chemical leaf traits, the climatic niche breadth of the tree species played an important role in mediating the interactions between herbivore and pathogen damage. Effects of herbivores on pathogens, and vice versa, were most pronounced for tree species with a narrow niche breadth. Climatic niche breadth might be a proxy for long‐term, evolutionary associations between plant species, and their consumer assemblages (Schuldt et al., 2012; Scriber, 2010). Plant species with a wider niche breadth probably have accumulated a more diverse set of consumer species over time (Lewinsohn et al., 2005; Miller, 2012), which could lead to higher consumer pressure and higher damage at local scales (Schuldt et al., 2012). Facilitative consumer effects might then be less relevant for tree species that are readily attacked by a diverse set of herbivores or pathogens. Tree species richness reduced pathogen damage particularly on tree species with such wide climatic niches. We speculate that an evolutionarily more stable host–pathogen relationship in these tree species (e.g., because wider climatic niches allowed the persistence of these relationships during past climatic changes, or because they allow for larger and less extinction‐prone pathogen populations; Schuldt et al., 2012) might have promoted host specialization of pathogen species (Brändle & Brandl, 2001). Highly specialized pathogens can be expected to respond particularly strongly to host dilution in species‐rich plant communities (Hantsch et al., 2014). Finally, environmental constraints might play a role in determining the effects of climatic niche characteristics. Pathogen and herbivore damages tended to increase with increasing niche marginality of the tree species. Plant species growing at their climatic range margins are often considered to be more susceptible to primary consumers because stressful environmental conditions could reduce their ability to cope with attacks (Fine et al., 2004; Maron & Crone, 2006).

### 4.2 Effects of herbivore and pathogen damage on tree growth

Despite the indications of potentially facilitative effects of herbivore and pathogen damage on each other and the modifying effects of tree species richness, we did not detect any significant interaction effects of herbivore and pathogen damage on relative tree growth rates. Additive effects of herbivores and pathogens on plant performance are often explained by the ability of plants to compensate or even overcompensate (i.e., showing increased performance) for the effects of tissue damage by different types of damaging agents (reviewed by Hauser et al., 2013). This means that reallocation of resources and increased growth, for example of less affected plant parts, might counteract potential interactive effects on plant growth and result in an additive net effect on growth at the level of the whole plant (Hauser et al., 2013). Interactions between herbivores and pathogens might therefore go unnoticed if they are not strong enough to result in detectable effects at the level at which plant performance is measured (in our case growth at the individual tree level). However, these patterns have not been explicitly addressed in the context of BEF research so far. Our study is the first, to our knowledge, to demonstrate that despite modifying effects of tree species richness on herbivore–pathogen relationships, the net effects of both damage types seem to be additive. Considering the increasing realization that higher trophic levels and their impact on producers play an essential role in modifying BEF relationships (Lefcheck et al., 2015; Soliveres et al., 2016), our study provides important insights that help to develop a clearer understanding of such effects in a BEF context.

Interestingly, RGRs in our study were positively related to herbivore damage, but negatively to pathogen damage. However, this was particularly true for smaller tree individuals. The effects of herbivory became increasingly detrimental (see also Schuldt et al., 2015) and the negative effects of pathogen damage became weaker with increasing tree size. This could be attributable to the tree size dependence of compensatory growth responses, where ontogenetic differences in resource storage and allocation can influence the ability of trees to compensate for leaf damage (Massad, 2013). These responses may differ among tree species, as shown by the modifying influence of morphological leaf traits on herbivore impact and of niche characteristics on the impact of both herbivores and pathogens. RGRs increased with herbivory for species with conservative resource use strategies (evergreen species with negative loadings on PC1 morph and positive loadings on PC2 morph), but not for less conservative species. However, conservative plant species usually show slower compensation rates following herbivory (Coley, Bryant, & Chapin, 1985; Nykänen & Koricheva, 2004). Cause and effect could therefore also be reversed in this case: Our results might reflect increased herbivore performance and damage on evergreen tree species with high RGRs, because investments in defense against damage can be greatly reduced in such cases (Coley et al., 1985; Endara & Coley, 2011). However, preliminary results from a manipulative study that used insecticides to exclude herbivores at our study site indicate that tree growth increases when herbivory is reduced (Y. Huang et al., unpublished data), supporting our expectation that herbivory influences tree growth.

Pathogen and herbivore effects on RGRs were also influenced by niche characteristics. Growth rates were negatively related to herbivory in species with narrow niche breadth, and positively in species with wide climatic niches. Similarly, negative effects of pathogen damage on growth rates were most pronounced for tree species growing at their climatic range margins. As discussed above, species with narrow niches and marginal populations might be more prone to experiencing stressful environmental conditions at our study site. Compared to more widespread species, this could impair their ability to (over‐)compensate for leaf damage effects (Wise & Abrahamson, 2007). Moreover, niche breadth interacted with tree species richness to affect RGRs. Positive effects of niche breadth on RGR were most obvious in monocultures, and became increasingly weaker in the more species‐rich mixtures. Species with narrow climatic niches might have less common niche characteristics (Slatyer, Hirst, & Sexton, 2013) that may lead to decreased performance when forced into growing in monospecific stands (Levine & HilleRisLambers, 2009). In contrast, species with wide niche breadth might exhibit more common niche characteristics that enable these species to cope with the specific conditions encountered in monocultures. Differences in climatic niche breadth might be less relevant for RGR in more diverse communities, because plot conditions mediated by the tree communities are so variable that specific demands are met more easily. However, further studies are needed to corroborate such a hypothesized modifying effect of niche breadth on BEF relationships.

It is notable that the effect of pathogen damage on tree growth became increasingly detrimental as tree species richness increased, despite the concomitant decrease in mean pathogen damage rates. Although we can only speculate on the underlying mechanism, previous studies have shown that while plant species richness reduced pathogen loads, it increased pathogen diversity (Hantsch et al., 2013; Rottstock et al., 2014). A higher pathogen diversity, with a higher probability of spillover of pathogen species from one tree species to another in mixtures with more tree species, could lead to a more diversified response (owing to a more diverse set of pathogens) and defense induction of plants (Spoel, Johnson, & Dong, 2007). It would be interesting to test whether the costs of such a response could result in negative effects on tree growth, despite a reduction in total damage levels.

The variation left unexplained by our models indicates that a large part of the variability in growth rates among trees depends on factors that act at the individual tree level (Fichtner et al., 2017). Many of our explanatory variables were plot‐ or species‐specific, meaning that we are unable to account for intraspecific trait variation that is influenced by environmental conditions and species richness (Li et al., 2017). Moreover, the precision of measurements of damage (six broad percentage classes) and growth (to the closest cm for height and mm for diameter) differed substantially and might have led to very conservative estimates of the relationships between damage and growth (also reflected by relatively low standardized estimates of some of the explanatory variables). Nevertheless, when looking at intraspecific and plot‐level patterns, the fixed factors in our models accounted for roughly 30%–50% of the variation attributed to random factors such as species identity and plots, which shows that our study covered very relevant factors. This means that the predictors identified by us will have important implications for community‐level effects, that is when considering the net effect of damage and species richness on stand‐level growth.

## 5 CONCLUSIONS

Patterns in real‐world systems and the net effects on ecosystem functions at the community‐level are determined by a multitude of pathogen, herbivore, and plant species. Our study considered the whole‐assemblage impact of insect herbivores and foliar fungal pathogens on tree growth. The results indicate that the assemblages of herbivores and pathogens have exclusively additive net effects on tree growth, although both damage types clearly affected each other. Our study is the first to show that these effects remain additive under scenarios of biodiversity loss. This is despite the fact that herbivore–pathogen relationships were influenced by tree species richness, which suggests that the latter can change the associations between different types of plant consumers. Moreover, our study highlights the usefulness of considering larger‐scale niche characteristics of the host species, because they provide extended insight into how interactions among organism groups, the effects of biodiversity loss, and the impact on ecosystem function might be modified under real‐world conditions.

## CONFLICT OF INTEREST

None declared.

## AUTHORS' CONTRIBUTIONS

AS, LH, AF, WH, GvO, and HB conceived the ideas and designed methodology. AS, LH, YL, and EW collected the data. AS analyzed the data and wrote the manuscript. All authors contributed critically to the drafts and gave final approval for publication.
