# Supplementary material for: Herbivore and pathogen effects on tree growth are additive, but mediated by tree diversity and plant traits
Source: Ecol Evol. 2017 Aug 11;7(18):7462–74. doi: 10.1002/ece3.3292 (PMC5606881; doi:10.1002/ece3.3292)
Supplement: Supplementary file 1 [file ECE3-7-7462-s001.docx]

**Supporting Information**

**Table S1.** Loadings and explained variance of principal components (PC) selected from PCA reduction analysis on morphological leaf traits (most influential variables in bold)

| *Trait* | *PC1* | *PC2* |
| --- | --- | --- |
| Leaf area | 0.34 | **-0.82** |
| Specific leaf area | **0.54** | 0.45 |
| Leaf dry matter content | **-0.50** | -0.30 |
| Leaf toughness | **-0.59** | 0.20 |
|  |  |  |
| Standard deviation | 1.46 | 1.02 |
| % variance explained | 0.54 | 0.26 |
| Cumulative % explained | 0.54 | 0.79 |

**Table S2.** Loadings and explained variance of principal components (PC) selected from PCA reduction analysis on chemical leaf traits (most influential variables in bold)

| *Trait* | *PC1* | *PC2* |
| --- | --- | --- |
| Leaf N content | **0.59** | -0.24 |
| Leaf C content | 0.00 | 0.36 |
| Leaf C:N ratio | **-0.54** | 0.35 |
| Leaf P content | **0.47** | 0.05 |
| Leaf phenolics content | -0.29 | **-0.57** |
| Leaf tannin content | -0.23 | **-0.61** |
|  |  |  |
| Standard deviation | 1.52 | 1.42 |
| % variance explained | 0.38 | 0.34 |
| Cumulative % explained | 0.38 | 0.72 |

**Table S3.** Minimum-adequate mixed-effects model for the effects of pathogen and herbivore damage, tree species richness, plant traits and plot characteristics on the relative tree growth rate (based on *tree height*) (R²m = 19.0%; R²c = 42.5%)

| *Predictor* | *Std. Est.* | *SE* | *DF* | *t* | *P* |
| --- | --- | --- | --- | --- | --- |
| (Intercept) | -1.20 | 0.04 | 59 | -29.8 | <0.001 |
| Site B | -0.15 | 0.04 | 310 | -3.8 | <0.001 |
| *Elevation (log)* | *-0.04* | *0.02* | *250* | *-1.9* | *0.055* |
| Slope (log) | 0.04 | 0.01 | 258 | 2.7 | 0.008 |
| Pathogen damage (log) | -0.04 | 0.01 | 10200 | -7.2 | <0.001 |
| Herbivore damage (log) | 0.02 | 0.01 | 10240 | 4.1 | <0.001 |
| *Tree species richness (log)* | *0.00* | *0.01* | *239* | *0.3* | *0.791* |
| Initial height (log) | -0.20 | 0.01 | 10110 | -35.0 | <0.001 |
| *PC1morph* | *-0.05* | *0.03* | *30* | *-1.5* | *0.146* |
| PC2morph | 0.05 | 0.03 | 32 | 2.1 | 0.047 |
| *Niche breadth (log)* | *0.03* | *0.03* | *33* | *1.0* | *0.328* |
| Niche marginality (log) | -0.07 | 0.03 | 31 | -2.6 | 0.014 |
| Pathogen damage:height | 0.01 | 0.00 | 10270 | 2.8 | 0.005 |
| Pathogen damage:niche breadth | 0.01 | 0.01 | 9688 | 2.4 | 0.017 |
| Pathogen damage:marginality | -0.03 | 0.01 | 10240 | -4.5 | <0.001 |
| Herbivory:height | -0.02 | 0.00 | 10260 | -4.0 | <0.001 |
| *Tree richness:PC1morph* | *0.01* | *0.01* | *2415* | *1.8* | *0.073* |
| Tree richness:niche breadth | -0.02 | 0.01 | 3169 | -3.6 | <0.001 |
| Pathogen damage:tree richness | -0.01 | 0.00 | 9447 | -2.9 | 0.004 |
| Herbivory:tree richness | 0.01 | 0.00 | 9378 | 3.0 | 0.003 |

Standardized parameter estimates (with standard errors, degrees of freedom, *t* and *P* values) are shown for the variables retained in the minimal model. Non-significant terms retained in the minimal models are italicized. Log-transformed predictors are indicated by (log). Colons indicate interactions between two predictors. PC1 and PC2 = scores of the first and second principal component of a PCA on morphological or chemical leaf traits (see Tables S1, S2)

**Table S4.** Minimum-adequate mixed-effects model for the effects of pathogen and herbivore damage, tree species richness, plant traits and plot characteristics on the relative tree growth rate (based on *basal area*) (R²m = 18.4%; R²c = 36.0%)

| *Predictor* | *Std. Est.* | *SE* | *DF* | *t* | *P* |
| --- | --- | --- | --- | --- | --- |
| (Intercept) | -0.78 | 0.04 | 40 | -21.3 | <0.001 |
| Slope (log) | 0.06 | 0.01 | 233 | 4.1 | <0.001 |
| Pathogen damage (log) | -0.04 | 0.01 | 9295 | -5.3 | <0.001 |
| Herbivore damage (log) | 0.02 | 0.01 | 9196 | 3.3 | <0.001 |
| *Tree species richness (log)* | -0.01 | 0.02 | 116 | -0.7 | 0.511 |
| Initial basal area (log) | -0.15 | 0.01 | 9031 | -20.1 | <0.001 |
| PC1morph | -0.07 | 0.03 | 32 | -2.1 | 0.045 |
| PC2morph | 0.10 | 0.03 | 32 | 3.7 | 0.001 |
| *PC1chem* | -0.11 | 0.03 | 31 | -3.2 | 0.003 |
| *Niche breadth (log)* | 0.04 | 0.04 | 35 | 1.0 | 0.326 |
| *Niche marginality (log)* | -0.06 | 0.03 | 32 | -2.1 | 0.042 |
| Pathogen damage:basal area | 0.02 | 0.01 | 9506 | 2.9 | 0.004 |
| Pathogen damage:PC1chem | -0.02 | 0.01 | 9109 | -2.6 | 0.008 |
| Pathogen damage:niche breadth | 0.01 | 0.01 | 8181 | 1.7 | 0.091 |
| Pathogen damage:marginality | -0.02 | 0.01 | 9282 | -2.1 | 0.034 |
| Herbivory:PC1morph | -0.02 | 0.01 | 9398 | -3.0 | 0.003 |
| Herbivory:PC2morph | 0.03 | 0.01 | 9292 | 5.1 | <0.001 |
| Herbivory:niche breadth | 0.03 | 0.01 | 9410 | 3.5 | <0.001 |
| Tree richness:basal area | 0.01 | 0.01 | 8038 | 1.8 | 0.067 |
| Tree richness:PC1morph | 0.02 | 0.01 | 1404 | 2.6 | 0.010 |
| Tree richness:niche breadth | -0.02 | 0.01 | 1764 | -2.5 | 0.013 |
| Pathogen damage:tree richness | -0.01 | 0.01 | 7623 | -2.0 | 0.044 |

Standardized parameter estimates (with standard errors, degrees of freedom, *t* and *P* values) are shown for the variables retained in the minimal model. Non-significant terms retained in the minimal models are italicized. Log-transformed predictors are indicated by (log). Colons indicate interactions between two predictors. PC1 and PC2 = scores of the first and second principal component of a PCA on morphological or chemical leaf traits (see Tables S1, S2)
